# The effects of direct current stimulation and random noise stimulation on attention networks

**DOI:** 10.1038/s41598-021-85749-7

**Published:** 2021-03-18

**Authors:** Alberto Lema, Sandra Carvalho, Felipe Fregni, Óscar F. Gonçalves, Jorge Leite

**Affiliations:** 1 Psychological Neuroscience Laboratory, CIPsi, School of Psychology, University of Minho, Campus de Gualtar, 4710-057 Braga, Portugal; 2 Spaulding Neuromodulation Center, Department of Physical Medicine and Rehabilitation, Spaulding Rehabilitation Hospital & Massachusetts General Hospital, Harvard Medical School, Boston, USA; 3 Proaction Laboratory, Faculty of Psychology and Educational Sciences, University of Coimbra, Coimbra, Portugal; 4 I2P-Portucalense Institute for Psychology, Portucalense University, Porto, Portugal; 5 Department of Education and Psychology, University of Aveiro, Campus Universitário de Santiago, Aveiro, Portugal

**Keywords:** Neuroscience, Cognitive neuroscience

## Abstract

Attention is a complex cognitive process that selects specific stimuli for further processing. Previous research suggested the existence of three attentional networks: alerting, orienting and executive. However, one important topic is how to enhance the efficiency of attentional networks. In this context, understanding how this system behaves under two different modulatory conditions, namely transcranial direct current stimulation (tDCS) and transcranial Random Noise Stimulation (tRNS), will provide important insights towards the understanding of the attention network system. Twenty-seven healthy students took part on a randomized single-blinded crossover study, testing the effects that involved three modalities of unilateral stimulation (tRNS, anodal tDCS, and sham) over the DLPFC, during the performance of the attention network test (ANT) in three different conditions: standard, speed and accuracy. Results showed that tRNS was able to increase attention during more complex situations, namely by increasing alerting and decreasing conflict effect in the executive network. Under the Speed condition, tRNS increased efficiency of the alerting network, as well as under the more demanding conflict network, tRNS overall increased the performance when comparing to sham. No statistical significant effects of tDCS were observed. These results are compatible with the attention requiring the synchronization of pre-existing networks, rather the reinforcement or creation of new pathways.

## Introduction

Attention is a complex core cognitive function, responsible for prioritizing the selection for further processing of internal and/or external sensory stimuli^1^. Attention can act through a bottom-up process in response to externally driven salient stimuli or by a top-down approach, guided by previous knowledge, planning and goals^2^. As a process, attention relies on multiple brain networks. According to Posner and Peterson^3,4^, attention encompasses three brain networks: alerting, orienting and executive. Alerting network is activated to maintain a state of readiness and is linked to thalamic and frontoparietal areas of the left hemisphere^5,6^. The orienting network is associated with spatial orientation, as well as to covertly direct attention to focus on specific stimuli^7^ and is linked to the activation of the frontal eye field (FEF) and superior parietal cortex, mainly on the right-hemisphere^3^. The executive control network refers to the process of conflict resolution associated with a goal^8^ and is dependent on the activation of the dorsal anterior cingulate (ACC) and dorsolateral prefrontal cortex (DLPFC)^8–11^. Although early studies suggested the independence of these three networks^8^, there are also reports of that the three networks rather act in an inter-dependent manner^12^, at least when participants are performing the attention network test (ANT).

The ANT^8^ is a task, in which several warning cues and flankers targets are combined, in order to probe the efficiency of each attention network, which has been widely used with healthy^8,13,14^ and clinical populations^15–17^. In each trial, 5 arrows are presented on screen and participants are required to make a left or right decision based on the direction that the middle arrow is pointing at. Flankers can point towards the same direction of the central arrow (i.e. congruent), or in the opposite direction (incongruent). And there can be also warning cues before the target, that could either be in the center, and thus replacing the fixator, above or below the fixator, or even double cues (i.e. above and below). This combination of no cues with spatial cues and congruent and incongruent flankers allows to test the three attentional networks^8^.

Interestingly enough attention is not a static process. Instead it relies on successful interregional communication^18^. Interregional correlation time series between brain regions, or functional connectivity (FC) showed that attention is a process involved in large scale brain network modulation, which includes within and between network modulation^19,20^, as well as reoccurring patterns of network modulation^21,22^. Thus, it is not surprising that these large scale FC modulations immediately preceding stimuli presentation are a predictor of subsequent response^23,24^. Moreover, by using a paced finger tapping paradigm, these moment-to-moment fluctuations in large scale brain networks were also shown to be significant for attention. For instance, spontaneous increase in tap variance (i.e. out of the zone) was associated with dorsal attentional and salience networks, while decreases in tap variance (i.e., in the zone) was associated with the default brain network^25^. This has been interpreted as being attentionally focused or in a mind wandering state^26^.

The dorsolateral prefrontal circuit is a suitable area for targeting attention enhancement, as it shares many connections with cortical and subcortical structures such as the caudate nucleus, globus pallidus, substantia nigra and the thalamus^10,27^. The DLPFC has an important role in top-down processing as well as inhibition of irrelevant stimuli^28^. Neuroimaging studies have shown greater activation of left hemisphere during tasks related to rapid changes as well as conflict resolution^3^. Namely, the left hemisphere seems more involved in attention cued paradigms whereas the right hemisphere seems more involved in slower responses over time, such as sustained attention^3,11^. Therefore, attention paradigms such as the ANT are more likely to involve left hemisphere activation, especially to attend to warning cues and conflict resolution among conflicting targets. Moreover, the left DLPFC has already been used as a target area for the study of attention in combination with transcranial electric stimulation (tES)^29,30^. And, as such attention has been a target for cognitive training alone in healthy^31^, or in clinical populations^32,33^.

Transcranial direct current stimulation (tDCS) is one of the most used tES techniques, which has been shown to increase working memory^34^, cognitive flexibility^35,36^, inhibition^37^, and attention^38^, among other cognitive functions. In tDCS, a weak electric current is delivered to the scalp that is able to induce neuromodulatory (i.e. change the likelihood of firing) and neuroplastic effects (by LTP and LTD)^39,40^. For instance, anodal tDCS over the right inferior frontal gyrus (i.e., 2 mA for 30 min) significantly increased performance in the alerting network^38^. Similarly, the orienting network has also been successfully modulated by anodal tDCS over the posterior parietal cortex (PPC) at 1.5 mA for 20 minutes^41^. Recently, offline anodal tDCS over the left DLPFC at 2 mA for 20 min showed a significant increase performance in the executive network^29^. Moreover, tDCS over the prefrontal cortex was able to increase performance in the orienting and executive networks in people with fibromyalgia^17^.

Another tES technique that can modulate large-scale networks^42^ is transcranial random noise stimulation (tRNS), a stimulation technique based on alternating current delivered at random normally distributed frequencies ranging from 0.1 to 640 Hz^43^. Terney and colleagues were among the first to demonstrate the tRNS excitatory effects on the motor cortex (M1)^44^. Since the first studies on motor cortex, tRNS has also proven to be effective in increasing performance in visual discrimination of faces^45^ and emotions^46^, numerical cognition, especially on complex scenarios^47^, as well as working memory^48^. Despite the fact that tRNS is able to modulate large-networks within the brain, and the fact that attention relies of several large scale networks in the brain, there was one previous attempt to study the effects of tRNS on affect, pain and attention was conducted on people suffering from multiple sclerosis^49^. However, the authors only showed that tRNS over the left dorsolateral prefrontal cortex (DLPFC) was only able to decrease pain. Thus, to the best of our knowledge, no previous studies have suggested specific frequency bands or applied full spectrum tRNS on attention, in healthy population.

Previous studies have shown that both tDCS^29,38^ and tRNS^47^ may actually improve performance, however potentially through different mechanisms such as creation of new connection or the development of new learning circuits in the case of tDCS, or better functional connectivity at critically across the pre-existent neural circuitry^50^. In this context, understanding how the attentional system would behave under two different modulatory conditions (tDCS and tRNS). would provide important insights towards the understanding of the attention network system. Therefore, the objective of this study was to study the effects of anodal tDCS and full spectrum tRNS on attentional network efficiency, as assessed by the attention network test (ANT).

## Methods

### Participants

A total of twenty-seven student volunteers, aged between 20 to 32 years old (M 22.78; SD 3.89; 7 males), recruited on campus, participated in this study (see Table 1). All participants reported to be healthy, with normal or corrected-to-normal vision and were right-handed (Edinburgh Handedness Inventory: EHI ≥ 80)^51^. Participants were excluded if they had any contraindication to receive tDCS/tRNS (such as metal in the head, implanted brain medical devices, scalp injuries prior experience of active tDCS/tRNS adverse effects, seizures, frequent headaches or migraines, use of medication, history of epilepsy, history of psychiatric/neurologic disorders, and any uncontrolled health conditions likely to worsen patient’s functional status in next 6-months such as cancer, terminal heart, kidney, or liver disease). Additionally, we excluded participants who reported extreme fatigue due to insufficient sleep in any stimulation session. All participants reported compliance with the experiment's initial recommendations to avoid alcohol, cigarettes and caffeinated drinks on the day of the experiment and none reported fatigue due to insufficient sleep. The study was performed in accordance with the Declaration of Helsinki, and all participants gave their written informed consent prior to their inclusion in the study. The study was approved by the local ethics committee*.*

**Table 1:** Sociodemographic and clinical information.

Stimulation	N	Age Mean (SD)	BDI Mean (SD)	STAI-Y state Mean (SD)	STAI-Y trait Mean (SD)
Female	19	22.16 (3.99)	4.11 (3.41)	33.68 (8.76)	35.42 (9.72)
Male	5	22.40 (2.51)	3.60 (3.78)	34.40 (9.13)	34.60 (8.29)
Total	24	22.21 (3.68)	4.00 (3.41)	33.83 (8.64)	35.25 (9.27)

### Design

#### Overall procedure

This is a randomized, single-blinded and crossover controlled study in which participants were randomized to receive both anodal tDCS, tRNS, and sham tDCS/tRNS. In order to decrease inter-individual effects, all participants received the 3 stimulation conditions. The stimulation order was fully randomized and counterbalanced across participants. The interval between each session was of, at least, 3 days to account for any carry-over stimulation effects^43^. We assessed the presence and severity of mood and anxiety symptoms in the initial screening using the Beck Depression Inventory—BDI^52^ and the State-Trait Anxiety Inventory—STAI-Y^53,54^. In the pre- and post-stimulation assessments, participants were screened about discomfort, fatigue, pain, itching, humor, tingling, burning, headache and sleepiness (among others) using a continuous Visual Analog Scale (VAS). Participants also responded to a modified version of the Positive and Negative Affect Schedule (PANAS)^55^ to assess changes in mood associated with the stimulation. At the end of each session, we assessed the blinding procedure's efficacy with a blinding questionnaire. The duration of each session was approximately 45 min.

**Figure 1:**
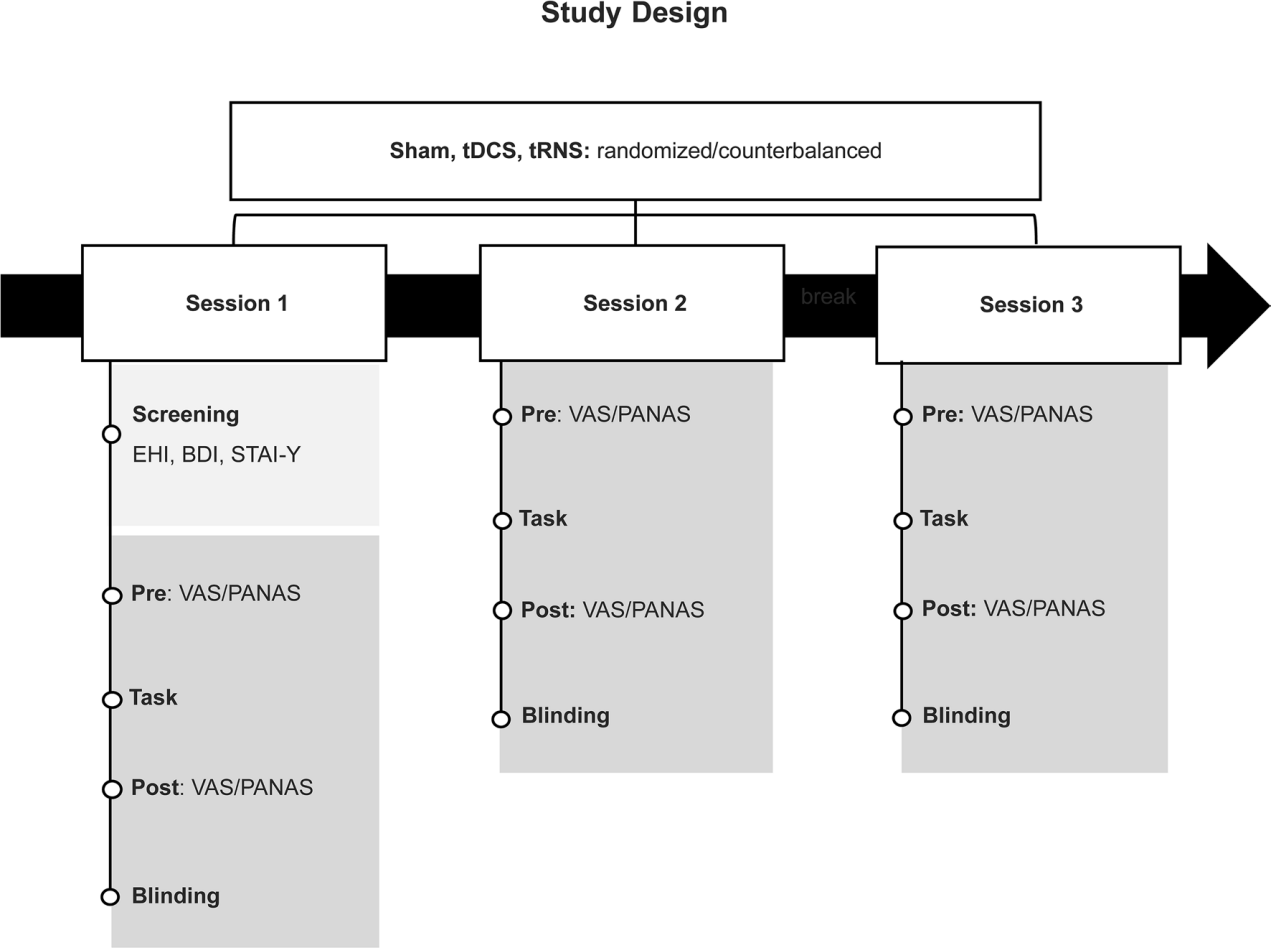
Study design. Cross-over controlled study with three experimental sessions: anodal tDCS, tRNS, and sham tDCS/tRNS randomized and counterbalanced across participants. The interval between each session was of, at least, 72 h.

Please see Fig. 1 for a schematic representation of the study.

### Experimental task

**Figure 2:**
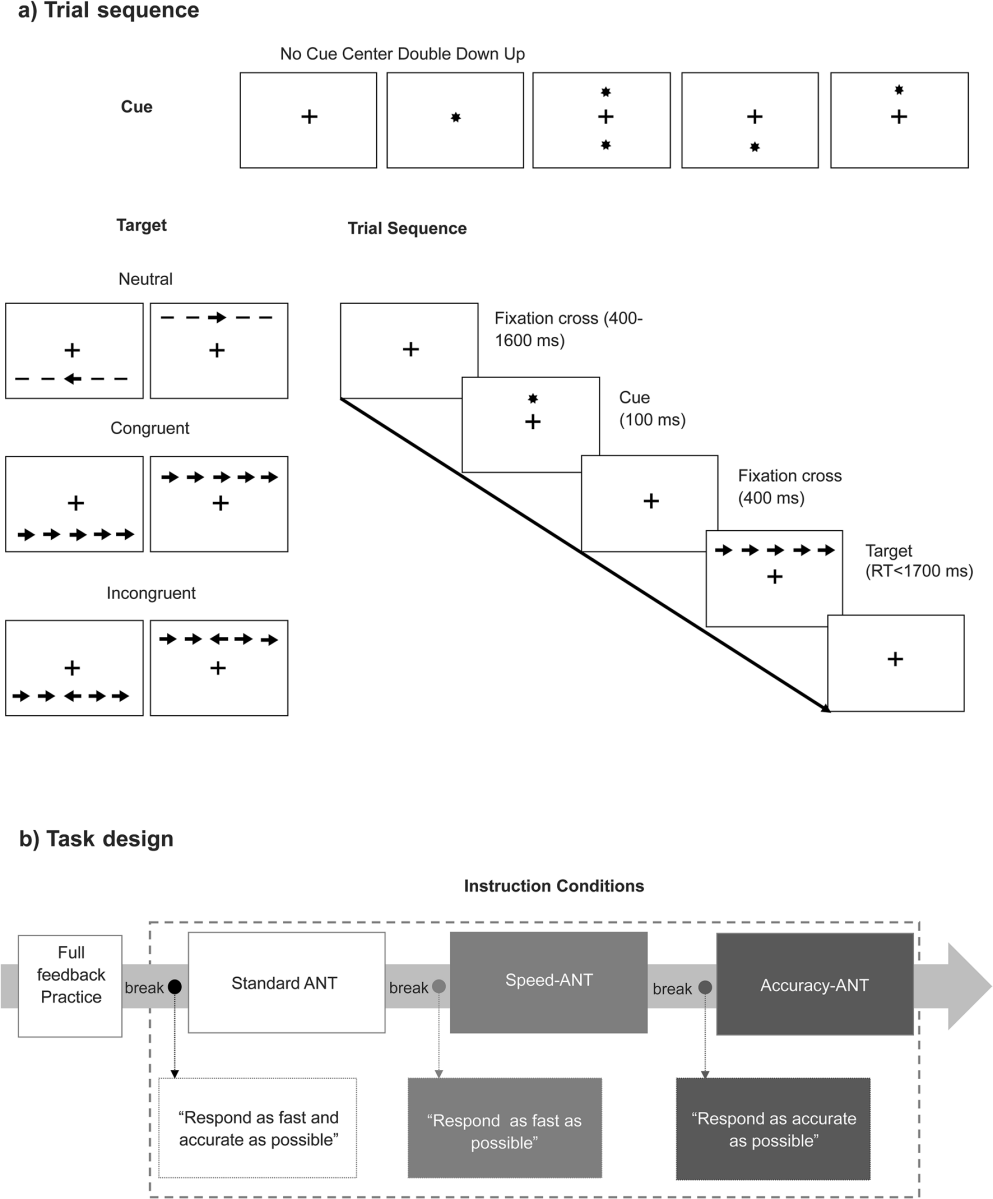
Attention network test (ANT) description and task design. (**a**) Cues, targets and trial sequence. (**b**) Task design: instructions were always given in the same order: first *Standard Condition* (regular task instructions), then, after a short break, *Speed Condition* (participants were asked to respond as fast as possible) and finally, after a new break, *Accuracy Condition* (participants were asked to respond as accurate as possible) followed.

We administered a modified version of the attention network test described by Fan et al.^8^. In this task, participants were asked to press a key indicating the direction (i.e., left or right) of a central arrow flanked by distracters pointing to the same or opposite direction (i.e., neutral “- -> - -”, congruent “ >  >  >  >  > ” or incongruent trials “ <  <  >  <  < ”)^56^. Before the target presentation, participants could be cued with an “*”, which could appear at an up, down or both (i.e., double) position from the central fixator, signaling a probable location of the following target or no cue (See Fig. 2A). Each trial consisted of a stochastic fixator period (400–1600 ms), a brief cue presentation(100 ms), a fixation period (400 ms) and a target presentation until participant response (max. 1700 ms), and an inter-trial interval with a variable duration, in order to ensure that each trial lasted for a total of 4000 ms (See Fig. 2).

The ANT had four blocks: a full-feedback 24 trials practice block and three experimental blocks. Each experimental block had 96 trials which were determined by: target directions (left, right) × target locations (up, down) × flanker congruencies (neutral, congruent, incongruent) × cue types (no cue, center cue, double cue and spatial cue), all repeated twice. Hence, 288 experimental trials were completed per session. The duration of the task was about 20 min. We modified the instructions of the ANT by presenting a new instruction before each experimental block, during the breaks. Instructions for each block followed a fixed sequence: (i) first experimental block, participants were given standard instructions to “respond as fast as possible, without committing errors” (i.e., Standard-ANT); (ii) second experimental block, participants were asked to “respond as fast as possible” (i.e., Speed-ANT); (iii) third block, instruction was changed to “respond as accurate as possible” (i.e., Accuracy-ANT). This was done in order to induce a change in participant´s response strategy, by increasing their focus on response speed or on accuracy. Before the beginning of each experimental block, participants were given a short break and the new set of instructions. The presentation order of the instructions was kept constant throughout all sessions and participants (i.e., Standard ANT, Speed-ANT, and Accuracy-ANT conditions) to allow for comparisons between blocks and stimulation duration (See Fig. 2B).

The ANT used in this study was programmed with E-Prime version 2.0 SP1 (Psychology Software Tools, Sharpsburg PA, US) and presented using a desktop computer running Windows 10 with a 17-in LCD (Fujitsu DVI-VGA) monitor with a 1280 × 1024 pixels’ resolution and a 75 Hz frame rate. Participants seated at approximately 60 cm of the screen and were required to respond by pressing the right or left mouse buttons.

### Stimulation protocols

**Figure 3:**
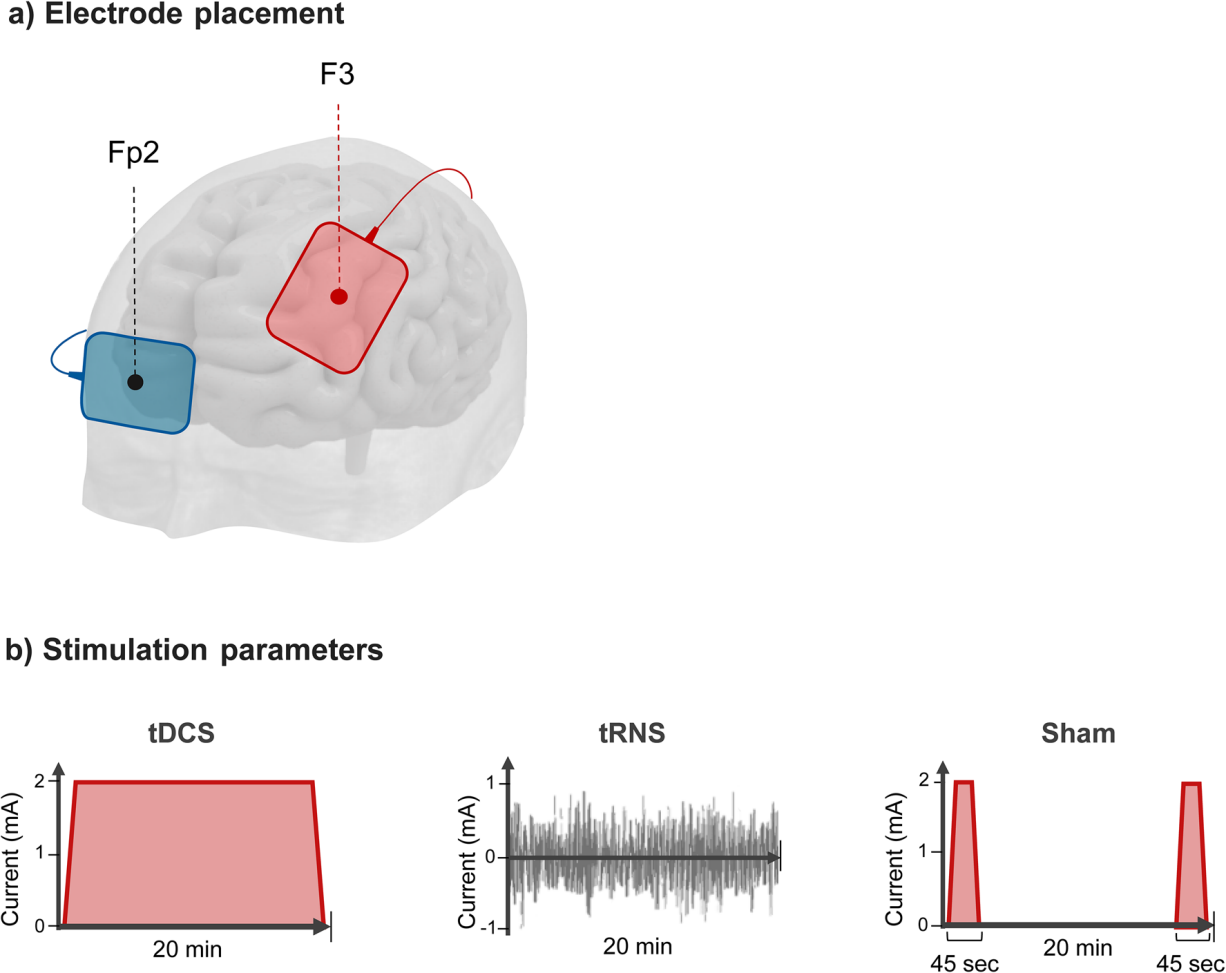
Electrode placement and stimulation parameters. (**a**) Electrode placement. 35 cm^2^ electrodes were used for both anode and cathode. Red dot represents the anode, over the left DLPFC (F3) while the blue dot represents the cathode, over the contra-lateral supra-orbital area (FP2). All stimulation conditions (sham, anodal-tDCS and tRNS) followed the same montage. (**b**) Stimulation parameters: tDCS for 20 min at 2 mA with a ramp in/out of both 15 s; tRNS current intensity was of 2 mA, with full-spectrum frequency 0.1–600 Hz; sham-tDCS was delivered at the beginning and at the end of 20 min applying 45 s of real stimulation (15 s ramp-up, 15 s stimulation and 15 s ramp-down).

Electric current was delivered by a 5 × 7 (35 cm^2^) cm pair of rubber electrodes in saline-soaked sponges placed over the left DLPFC (F3; anode) and the contralateral supraorbital area (Fp2; cathode), in accordance to the International 10–20 EEG System^57^ (See Fig. 3A). 2 mA of anodal tDCS was delivered for 20 min (with 15 s of ramp in/out). tRNS was delivered at 2000 μA with full-spectrum frequencies ranging from 1 to 640 Hz, for 20 min. Both stimulations were delivered continuously for 20 min during the completion of the task. Sham-tDCS consisted of 45 s of stimulation delivered at the beginning and at the end of the 20 min (i.e., 15-s for each phase: ramp-up, stimulation, and ramp-down). We applied this blinding procedure, with 15-s of *verum* stimulation in the beginning and in the end, in order to minimize the difference between the sensations induced by the active stimulation (especially the tRNS) when compared to the sham condition (see Fig. 3B). Previous studies have shown that the blinding may be compromised in repeated measures designs with a high current intensity such as 2 mA^58–60^. All stimulation techniques were applied using a Magstim Eldith DC Stimulator Plus (Neuroconn, DE).

### Assessments

The Edinburgh Handedness Inventory—EHI^51^ was used to measure hand preference for common manual tasks. Participants are asked to rate 10 statement on the use of the right or left hand when performing a specific action (e.g., writing or using scissors). Scores range from − 100 to 100 for left and right-handed, respectively. To be included as right-handed in the study, participants needed to score above 80.

To measure the presence and severity of depressive symptoms across affective, cognitive, motivational, and functional domains, we used the Beck Depression Inventory—BDI^52^. The BDI is a 21-item rating inventory to assess how the subjects feel “at that moment” about each statement on a four-point scale (symptoms severity increases from 0 to 3). Participants with scores above 9 (i.e., symptomatic), were excluded due to known effects of depression on cognition and reaction times.

State-Trait Anxiety Inventory—STAI-Y^53,54^ is a 40-item scale used to measure trait anxiety (e.g., “I worry too much over something that really does not matter”) and state anxiety (e.g., “at that moment” anxiety feelings such as “I am tense”). Items are rated on a four-point scale, with higher ratings indicating greater anxiety. Scores for each subtest ranges from 20 to 80 with a cut-off score of 40 for clinically significant anxiety symptoms.

The Positive and Negative Affect Scale—PANAS^55^ is a 20-item scale that measures the affective disposition composed by two mood scales: positive (PA) and negative affect (NA). PA is defined as activation or engagement and is represented by positive mood items like “Interested” and “Excited.” In contrast, NA is defined by lethargy or sadness and is represented by negative mood items like “Irritable” and “Afraid”. Participants are asked to rate the extent to which they experience each item within a specified period (e.g., “right now” or “over the past week”) on a five-point scale (from 1—Very slightly or not at all to 5—Extremely). Scores range from 10 to 50 for each subscale, with higher scores indicating higher PA or NA levels.

The Visual Analog Scale/tES—VAS. The VAS is a 10-item scale that measures potential adverse effects of tES on a continuous scale (from 0 to 10). Adverse effects are assessed on several domains: tiredness, anxiety, sadness, agitation, sleepiness, itch, headache, pain, tingling, and metallic taste. This scale was administered before and after the intervention to control for potential adverse effects of the stimulation.

Adult Safety Screening Questionnaire. This questionnaire was administered to determine the participants' suitability to undergo tDCS and tRNS interventions. It is a 16-item instrument that assesses the prior contact with tDCS or other tES techniques, past adverse effects, occurrence of convulsions, stroke, serious head injury, frequent or severe headache, implanted medical devices, medication, pregnancy and epilepsy. All items are answered by a yes/no. If yes to any of the items, more detailed information was asked.

We used a blinding Questionnaire to measure the efficacy of the blinding procedure, as perceived by the subjects. Participants were asked to indicate if they think the tDCS/tRNS intervention was active, sham, or do not know the answer. Additionally, they were required to mention how confident they feel about their response on a five-point scale (from not confident at all to extremely).

### Data analysis

All data analyses were performed using SPSS version 25 (IBM, United States). From the 27 participants enrolled in the study, a total of three participants were excluded. Two of them were excluded due to the presence of psychiatric symptoms as indexed by BDI scores of 13 and 14 and one of them due to high scores in VAS (i.e., > 8) for fatigue and anxiety across all sessions. Therefore, final analyses were performed with 24 participants.

Prior to computing the network scores from the ANT, we selected only correct responses, resulting in a loss of 1.98% of the data. We also removed outliers with response time (RT) < 200-ms and > 1200-ms, which ended up in an additional loss of 0.28% data. The remaining data base was used for both RT and Accuracy analyses. For RT, we calculated the ANT scores using the median of correct responses as a measure of central tendency^61,62^. We calculated all networks scores according to Westlye and colleagues^62^ formula, by subtracting the RT from relevant network-conditions and scaled to percentage by dividing the network score by the center cue (alerting network), spatial cue (orienting network) and congruent target (executive network). Please see the following formulas for more details on the percentage scaled results.

$$ Alerting = \frac{{RT\left( {no cue} \right) - RT\left( {center} \right)}}{{RT\left( {center} \right)}}, $$

$$ Orienting = \frac{{RT\left( {center cue} \right) - RT\left( {spatial} \right)}}{{RT\left( {spatial} \right)}}, $$

$$ Executive = \frac{{RT\left( {incongruent} \right) - RT\left( {congruent} \right)}}{{RT\left( {congruent} \right)}}. $$

For alerting and orienting ANT scores, higher scores indicate better performance (i.e., benefits from cue presentation), whereas for executive network ANT scores, lower scores indicate better performance (i.e., less cost related to the filtering of incongruent target). We performed an additional analysis on RT of no cue condition under standard condition to verify the effects of the stimulation techniques at a motor level. For accuracy, we computed the mean percent of correct response (ACC) of the cues involved in each network.

The statistical analyses for both RT and ACC followed these steps: normality of the distribution was assessed by Kolmogorov–Smirnov test; main analyses consisted of GLM repeated measures ANOVAs with instruction condition (standard, speed, accuracy) and stimulation condition (sham, tDCS, tRNS) as within-subject factors; data sphericity was assessed by the Mauchly test and corrected using Greenhouse–Geisser when appropriate; post-hoc comparisons were performed using LSD whenever significant effects were found (p < 0.05). As indexed by PANAS and VAS before and after stimulation scores, adverse effects were computed with paired-samples t-tests.

### Ethical approval and informed consent

All procedures performed in the study were in accordance with the ethical standards of the Declaration of Helsinki (1964). All participants gave written informed consent before they participated in the study. The study was approved by the local ethics committee, Ethics Committee for Research in Life and Health Sciences (CEICVS), at Minho University.

## Results

### Attention network test

#### Response time

**Figure 4:**
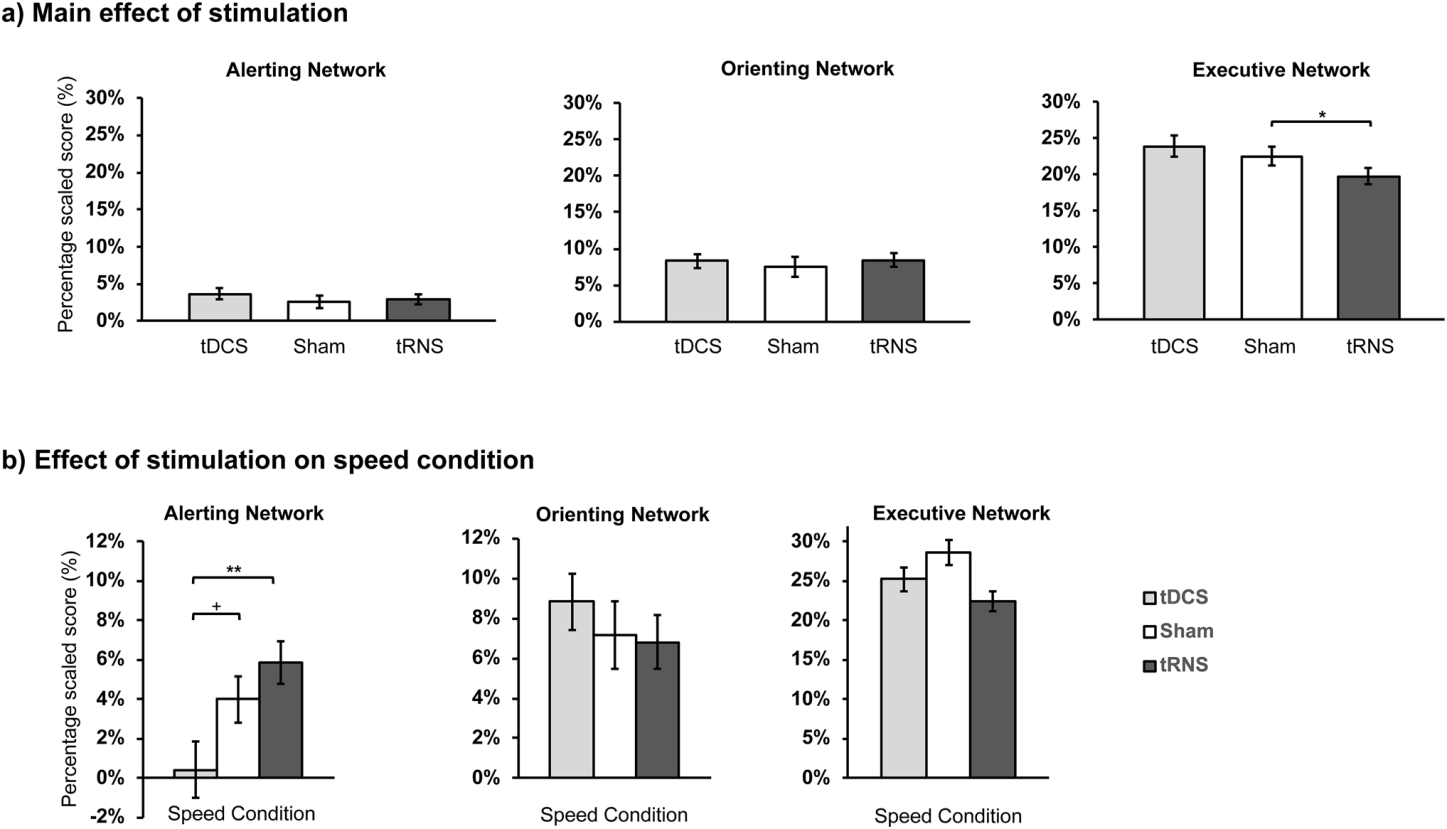
Response time effects on all attention networks. (**a**) Stimulation main effect for all attention networks. Only executive network showed a significant effect of tRNS improving conflict resolution when compared to sham (p = 0.022). (**b**) Interaction effects for all attention networks. Alerting network showed a significant interaction effect, namely, under Speed Condition, tRNS showed a facilitating effect when compared to tDCS (p = 0.005) but not compared to sham (p = 0.055). *p < 0.05 Error bars show Standard Error. ^+^p < 0.10, *p < 0.05, **p < 0.01, ***p < 0.001.

For the alerting network, the main analysis showed that there was no statistical significant effects for condition [*F*(2, 46) = 0.22, *p* = 0.801, *η*_*p*_^2^ = 0.010] or stimulation [*F*(2, 46) = 0.67, *p* = 0.517, *η*_*p*_^2^ = 0.028]. However, there was a statistically significant interaction effect between condition and stimulation [*F*(4, 92) = 3.53, *p* = 0.010, *η*_*p*_^2^ = 0.133]. Pairwise comparisons showed that under speed condition, tRNS increased ANT score as compared to anodal tDCS (*p* = 0.005), but not to sham (*p* = 0.330). Anodal tDCS marginally decreased the ANT score when compared to sham (*p* = 0.055) (see Fig. 4B).

For the orienting network, the main analysis showed that there was no statistically significant effect for condition [*F*(2, 46) = 0.33, *p* = 0.716, *η*_*p*_^2^ = 0.014], stimulation [*F*(2, 46) = 0.29, *p* = 0.744, *η*_*p*_^2^ = 0.013], nor interaction [*F*(4, 92) = 0.66, *p* = 0.616, *η*_*p*_^2^ = 0.028].

For the executive network, analyses showed significant effects for the condition [*F*(2, 46) = 12.20, *p* < 0.001, *η*_*p*_^2^ = 0.347] and stimulation, [*F*(2, 46) = 3.72, *p* = 0.032, *η*_*p*_^2^ = 0.139], yet no interaction effect was found [*F*(4, 92) = 1.29, *p* = 0.281, *η*_*p*_^2^ = 0.053]. For the condition main effect, as expected, speed condition increased the ANT score when compared to standard (*p* < 0.001) and accuracy conditions (*p* = 0.001), resulting in worse RT performance. For stimulation main effect, tRNS showed a decrease in the ANT score when compared to sham (*p* = 0.022), thus improving efficiency of the executive network by reducing the time needed to solve the conflict between congruent and incongruent target (see Fig. 4A and Table 2 for scores). Other comparisons were not statistically significant.

**Table 2:** RT and ACC for each attention network effects by instruction condition and stimulation.

Attention effect	Instruction condition	Stimulation					
		Sham (*N* = 24)		a-tDCS (*N* = 24)		tRNS (*N* = 24)	
		RT Mean (SD)	Acc %	RT Mean (SD)	Acc %	RT Mean (SD)	Acc %
Alerting	Standard	20.94 (30.34)	98.00 (3.16)	17.25 (38.05)	97.57 (3.45)	6.88 (27.50)	98.61 (1.90)
	Speed	17.19 (37.65)	95.14 (5.12)	0.17 (26.99)	97.22 (2.92)	27.06 (31.66)	97.48 (3.24)
	Accuracy	13.83 (32.14)	98.35 (1.37)	19.75 (40.27)	97.83 (3.71)	5.54 (28.48)	98.18 (2.90)
Orienting	Standard	42.54 (40.01)	98.18 (2.63)	32.31 (33.52)	98.00 (2.97)	42.08 (32.19)	98.70 (1.82)
	Speed	31.56 (42.31)	95.31 (5.18)	40.73 (37.44)	97.40 (3.89)	29.58 (37.51)	97.57 (3.45)
	Accuracy	34.52 (37.04)	98.52 (1.56)	43.77 (33.18)	98.26 (3.67)	44.00 (28.45)	98.87 (2.13)
Executive	Standard	99.17 (41.24)	97.92 (3.65)	100.85 (35.87)	96.81 (4.18)	92.29 (38.53)	98.24 (1.80)
	Speed	133.67 (60.21)	93.75 (6.65)	117.46 (36.66)	95.96 (4.54)	104.25 (42.73)	96.55 (4.22)
	Accuracy	116.25 (33.75)	97.92 (1.88)	105.85 (47.04)	97.07 (4.43)	89.54 (39.12)	97.59 (3.26)

Attention networks effects are derived from the ANT.

Additionally, we analyzed no cue RT as a probe for possible changes at the motor level. We analyzed the effects of stimulation condition on no cue RT under standard instruction using one way repeated measures ANOVA. Results showed no effect of stimulation [F(2, 46) = 2.33, p = 0.108, η_p_^2^ = 0.092]. Therefore, stimulation conditions did not change the RT of participants when no cue were presented. We also highlight in the discussion that the effects may be also due to motor components related to task performance, however, the data from the simple RT does not suggest that these effects are purely motor.

#### Accuracy: ACC

**Figure 5:**
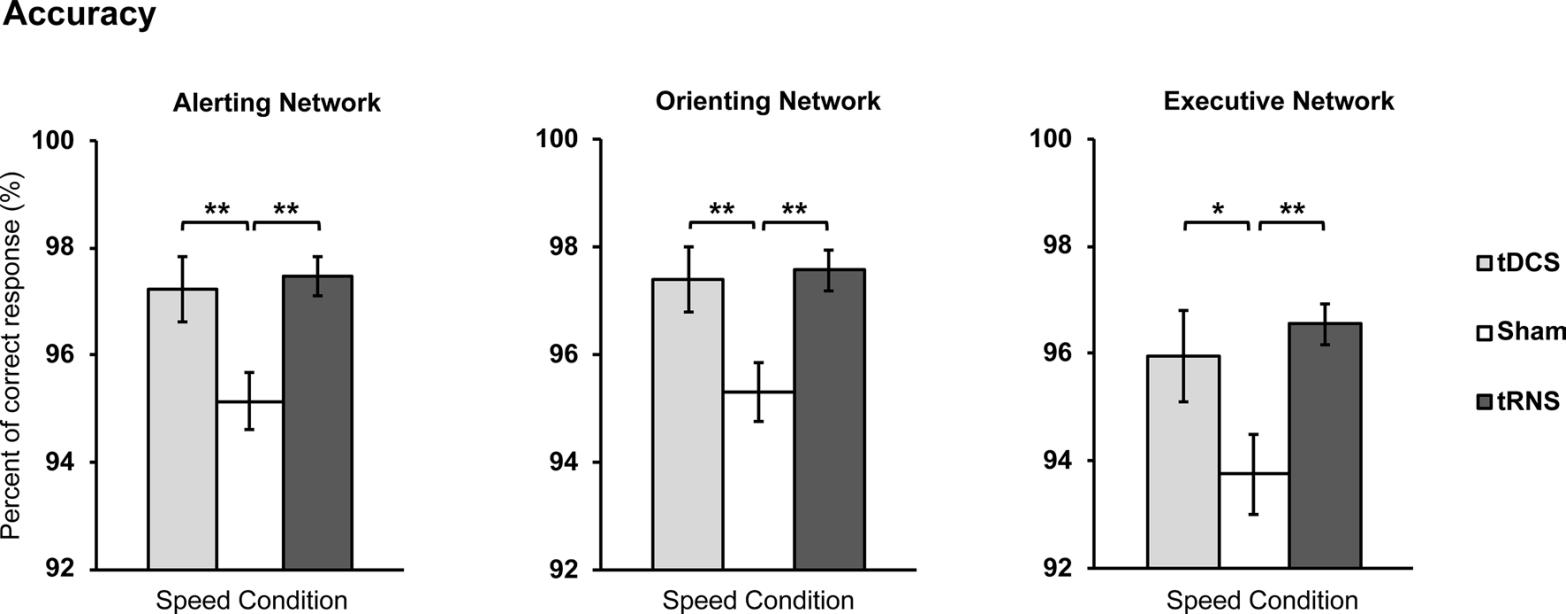
Accuracy effects on all attention networks under speed condition. Under speed condition (“respond as fast as possible”), tDCS and tRNS significantly improved mean percent of correct response when compared to sham across all attention networks. Error bars show Standard Error. *p < 0.05, ** p < 0.01, ***p < 0.001.

For Alerting, analyses showed that there was a statistical significant main effect for condition [*F*(1.49, 34.48) = 6.46, *p* = 0.008, *η*_*p*_^2^ = 0.219, ɛ = 0.75], but not for stimulation [*F*(2, 46) = 2.58, *p* = 0.086, *η*_*p*_^2^ = 0.101]. An interaction effect was also found [*F*(4, 92) = 2.96, *p* = 0.024, *η*_*p*_^2^ = 0.114]. Post hoc analysis for the interaction effect revealed that under the Speed condition, both active tRNS (*p* = 0.003) and tDCS (*p* = 0.008) showed a significant increase in accuracy when compared to sham (see Fig. 5; see Table 3 for more information). Other comparisons were not statistically significant.

**Table 3:** Mean (SD) RT and ACC for each cue and target by instruction condition and stimulation.

Stimulation	Cues and targets	Instruction condition					
		Standard		Speed		Accuracy	
		RT (M, SD)	Acc (%, SD)	RT (M, SD)	Acc (M, SD)	RT (M, SD)	Acc (M, SD)
Sham	No cue	554.50 (79.91)	99.61 (2.92)	521.04 (58.85)	96.01 (4.68)	556.58 (60.88)	98.61 (2.35)
	Center cue	533.56 (75.14)	97.40 (4.73)	503.85 (69.48)	94.27 (6.93)	542.75 (68.68)	98.09 (2.74)
	Double cue	526.23 (69.24)	98.96 (3.53)	510.46 (67.22)	95.66 (7.00)	541.90 (69.69)	97.92 (3.25)
	Spatial cue	491.02 (61.89)	98.96 (1.84)	472.29 (70.65)	96.35 (5.26)	508.23 (73.87)	98.96 (1.84)
	Congruent	498.15 (71.62)	99.61 (1.06)	466.02 (60.34)	99.48 (1.19)	504.77 (58.96)	100.00 (0.00)
	Incongruent	596.31 (87.98)	96.22 (7.37)	599.69 (98.78)	88.02 (12.52)	621.02 (79.65)	95.83 (3.76)
a-tDCS	No cue	529.31 (66.55)	97.40 (4.22)	515.19 (51.85)	97.92 (3.25)	562.17 (67.09)	97.57 (4.58)
	Center cue	512.06 (61.56)	97.74 (3.68)	515.02 (66.20)	96.53 (4.86)	542.42 (65.13)	98.09 (3.47)
	Double cue	513.06 (57.39)	97.57 (3.67)	506.25 (60.15)	96.18 (5.20)	540.54 (59.68)	97.40 (2.96)
	Spatial cue	476.25 (58.70)	98.26 (3.23)	474.29 (59.18)	98.26 (3.67)	498.65 (59.83)	98.44 (4.57)
	Congruent	478.13 (53.46)	99.22 (2.11)	472.46 (52.65)	99.61 (1.40)	510.63 (60.78)	99.48 (1.19)
	Incongruent	578.98 (74.50)	94.40 (8.54)	589.92 (57.76)	92.32 (8.19)	616.48 (76.09)	94.66 (8.37)
tRNS	No cue	532.15 (60.10)	98.78 (2.29)	524.75 (62.97)	97.92 (3.25)	540.44 (57.09)	97.92 (3.48)
	Center cue	525.27 (68.30)	98.44 (2.40)	497.69 (66.37)	97.05 (4.16)	534.90 (59.88)	98.44 (3.21)
	Double cue	519.58 (58.11)	98.61 (2.35)	498.81 (61.51)	97.05 (4.34)	532.90 (53.52)	97.22 (3.82)
	Spatial cue	483.19 (57.22)	89.96 (2.22)	468.10 (70.78)	98.09 (3.47)	490.90 (58.63)	99.31 (1.59)
	Congruent	488.75 (55.65)	100.00 (0.00)	468.44 (60.27)	99.74 (0.88)	502.75 (52.28)	99.87 (0.64)
	Incongruent	581.04 (83.41)	96.48 (3.61)	576.71 (66.77)	93.36 (8.56)	592.29 (61.04)	95.31 (6.58)

Orienting network analyses showed statistically main effects of condition [*F*(1.41, 32.46) = 5.81, *p* = 0.013, *η*_*p*_^2^ = 0.202, ɛ = 0.70] and stimulation [*F*(2, 46) = 4.67, *p* = 0.014, *η*_*p*_^2^ = 0.169], as well as an interaction effect [*F*(2, 46) = 2.86, *p* = 0.028, *η*_*p*_^2^ = 0.111]. The interaction effect showed that under Speed Condition, both active tRNS (*p* = 0.002) and tDCS (*p* = 0.001) increased accuracy when compared to sham (see Fig. 5; see Table 3 for more information).

For the executive network, analyses showed a statistically significant main effect for condition [*F*(1.34, 30.98) = 7.70, *p* = 0.005, *η*_*p*_^2^ = 0.251, ɛ = 0.67]; but not for the stimulation conditions [*F*(2, 46) = 3.02, *p* = 0.059, *η*_*p*_^2^ = 0.116].An interaction effect was also found [*F*(2.90, 66.77) = 4.09, *p* = 0.0115, *η*_*p*_^2^ = 0.151, ɛ = 0.72]. Post hoc analysis for the interaction effect revealed that under the Standard condition, active tDCS showed a decrease in conflict accuracy when compared to sham (*p* = 0.035) and tRNS (*p* = 0.038). Under Speed Condition, however, both active tRNS (*p* = 0.003) and tDCS (*p* = 0.012) increased conflict accuracy when compared to sham. Other comparisons were not statistically significant (see Fig. 5; see Table 3 for more information).

##### Exploratory analysis based on the effects of instruction blocks on accuracy

Post-hoc tests for the condition main effect did reveal statistically significant decrease in accuracy during Speed when compared to Standard (*p* > 0.001) and to Accuracy conditions (*p* = 0.019) in the alerting network. Post-hoc analyses for condition main effect for the orienting network showed that under Speed Condition, accuracy decreased significantly when compared to Standard (*p* = 0.006) and Accuracy conditions (*p* = 0.022). Similarly, for the executive networks, post hoc tests for condition main effect did reveal statistically significant decrease in accuracy when Speed Condition was compared to Standard Condition (*p* > 0.001) and to Accuracy Condition (*p* = 0.019).

### Adverse effects

Regarding the effects of each stimulation condition in affect (positive or negative) as assessed by the PANAS, there was no statistically significant difference in any stimulation condition (all *P*’s > 0.083). Results for adverse effects as indexed by VAS scores showed a statistically significant increase in itching [*t*(23) = 3.49, *p* = 0.002)] and tingling [*t*(23) = 2.37, *p* = 0.026], for anodal tDCS when comparing before and after stimulation. For tRNS, we only found a statistically significant increase in headache [*t*(23) = 2.18, *p* = 0.039]. As expected, anodal tDCS produced the highest itching score (See Table 4). However, it is important to highlight that these effects were mild, not averaging 3 on a 10-point scale. No phosphenes were reported by participants.

**Table 4:** VAS self-report differences before and after experimental protocol.

Stimulation	Dimensions	Before (N = 24) Mean (SD)	After (N = 24) Mean (SD)	*t* (23)
Sham	Itching	0.39 (1.03)	1.17 (1.53)	2.27*
	Tingling	0.00 (0.00)	0.35 (0.65)	2.58*
a-tDCS	Itching	0.58 (1.38)	2.21 (2.57)	3.49**
	Tingling	0.08 (0.28)	0.79 (1.41)	2.38*
	Headache	0.46 (1.06)	0.75 (1.11)	2.07^+^
tRNS	Itching	0.25 (0.85)	0.88 (1.65)	1.97^+^
	Headache	0.63 (1.38)	1.17 (2.06)	2.18*

Visual analogue scale (VAS). We only report symptoms that present a statistically significant increase between before and after the sessions (all symptoms assessed: tiredness, anxiety, sadness, agitation, sleepiness, itching, headache, pain, tingling and metallic flavor).

^+^*p* < 0.10, *p < 0.05, **p < 0.01, *p < 0.001.

### Blinding efficacy

In order to assess if participants were able to correctly guess the stimulation condition to which they were subjected to in that particular session (i.e., active or sham), participants responses about their guessed allocation were asked at the end of each session. Participants were able to correctly guess their allocation to sham and active conditions in 38 out of 72 sessions, which translates to 52.78%. Most of participants rated their confidence level on the responses as moderate (34.21%) or considerable (31.58%).

## Discussion

The aim of the present study was to examine the effects of tRNS and anodal tDCS over the left DLPFC, as compared to sham stimulation, on the alerting, orienting and executive networks of attention. Additionally, in order to probe potential effects on the ANT, we used three distinct blocks: standard, focus on speed and focus on accuracy.

Under the speed condition, participants who received tRNS performed better in terms of the alerting network ANT score by responding faster, when compared to tDCS. It has already been shown that tRNS applied to the visual cortex enhances perception^63,64^, or affects the binocular rivalry phenomenon^65^. As attention requires a transient long phase synchronization in the theta band between fronto-parietal-temporal regions^66^, intra and inter-regional modulation seems to be required in order to modulate attention. As synchronization between regions depend on several factors such as frequency, anatomical distances, axon conduction velocity, among others, effective communication requires a favorable signal to noise ratio. However, not all the neurons that are responsible for signal transmission will reach a threshold that will allow them to depolarize. In this sense, random noise stimulation may help to improve inter-regional transmission by the mechanisms of stochastic resonance^64,67^, in which the noise added to neural processing improves the signal in the attention network. For instance, using a global motion task, it has been suggested that high frequency tRNS was able to tune in neurons near the directional signal and improved signal pooling of the local cues, by an increased signal-to-noise ratio, which in turn increased the overall sensitivity for the global motion^68^.

On linear systems, noise induction impairs performance, however in non-linear systems, adding noise can actually increase performance^69^. Adding a subthreshold noise to a weak signal can increase its detection^44,67^, especially because stochastic resonance has been shown to modulate intra and inter regional neural synchronization^63,65,67,70^. This is especially relevant for tasks in which the synchronization of different brain regions is required, such as in the attention system. Therefore, it is possible that under the speed condition, increased network demands in the brain (which is a non-linear system), allowed for additional neurons to tune in by the mechanism of stochastic resonance, thus increasing performance in the alerting network.

Our exploratory analysis of accuracy suggests that speed emphasis actually decreased accuracy, in what is called a speed accuracy tradeoff (SAT). Moreover, compared to other conditions, the speed condition significantly increased the conflict effect in the executive network, thus, resulting in participants adopting a different response criterion under a time-constraint speed instruction (i.e., “Respond as fast as possible”). Therefore, the condition changed participants’ behavior.

First of all, it is important to explain this SAT process^71^. Typically, when speed of response is favored, accuracy is decreased, whereas, when accuracy is favored, a decrease in speed is expected. More than changes in participant’s strategy, neuroimaging studies suggest that SAT is mediated by an interplay between cortical and subcortical structures^72–75^. Namely, SAT seems to rely on cortical integrators^76^ (e.g. pre-supplementary motor area) and at the subcortical level through basal ganglia inhibitory activity^75,77^. Under speed it is possible that these cortical integrators receive additional excitatory inputs, which results in increased baseline activity; or that increases in striatal activity will decrease the inhibitory control of the basal ganglia over the brain^73^.

These theories highlight the importance of the relationship between cortical and subcortical regions. Regardless of the fact that the present study cannot provide insights about how the brain changes during the SAT, it is important to highlight that tDCS and tRNS had very distinct effects on the ANT scores, especially under speed instructions. Moreover, according to the speed-accuracy tradeoff, in the condition in which speed is favored, accuracy is decreased^71^. In other words, it is harder to maintain accuracy, with shorter response times. In this sense, with a focus on response time, accuracy decreases, simply because it is ubiquitously harder to maintain accurate performance (as demonstrated by the present data on the accuracy under the speed condition).

tRNS seems to have counteracted this increased difficulty demand over the network and, indeed, improved RT when the task complexity increased—especially on the case of the executive network. This phenomenon has already been shown before^47,63,78^. For instance, beneficial effects of tRNS during an arithmetic learning task have been shown previously, but only on the difficult condition when the number of repetitions was lower^47^. Moreover, random noise applied to the earlobes was also able to induce an effect on an arithmetic task, but only for the complex version^50^. More specifically, tRNS improved participant´s performance by decreasing response time for incongruent targets for the executive network. It is important to highlight that the executive network is the most demanding in terms of the cognitive resources required to perform the ANT, as it requires the filtering of task conflicting stimuli. In this sense, it is possible that tRNS may improve the filtering of relevant and irrelevant information related to the task^79,80^. Speed further increases cognitive demands due to the need to adjust the response threshold to faster responses when less information is available to make a decision, which has already been shown to induce greater activation in frontal regions, such as striatum, supplementary motor area (pre-SMA) and DLPFC^73,77,81^. As these changes rely on more efficient intra and inter regions communication, the facilitatory effects of tRNS may have occurred through subthreshold stochastic resonance, in which the added random noise to the neural system may have improved the detection of the signal by facilitating its processing and detection at critically^44,82^. According to the at critical brain hypothesis, brain networks operate near phase transition called at criticality, which lies between states of increased or decreased activation and therefore is not unique^83^. In this context, the presence of non-zero noise will add a subthreshold noise to the signal and, therefore, facilitate its transmission by reaching the at criticality point^84^. In this at critically point there is increased similarity between the input signal and the one that travels throughout the system, which can result, for instance, in increased target detection^64^. In this sense, tRNS was able to increase overall performance over the executive network by increasing the similarity between the input and the signal throughout the task related network, due to the stochastic resonance phenomenon^70,85^. However, this hypothesis of stochastic resonance should be further explored, especially by assessing stimulation-induced changes in the spontaneous brain oscillatory activity, as well as in the intra and inter-regional functional connectivity.

Anodal tDCS did not showed any effect on executive network, as previously found by Miler and colleagues in a similar experiment^29^. The instruction conditions in our study may have negatively affected tDCS ANT score due to changes in participants strategy as well as functional connectivity demands as shown by a decrease in accuracy on the executive network under the standard instructions. tDCS effects on attention may require constant instruction conditions or the combination with cognitive training to increase the efficiency in the orienting or executive networks^17^. This is compatible with the rationale underlying the effects of tDCS, namely modulation of cortical activity and excitability, which leads to secondary changes to synaptic connectivity (i.e., new learning)^86,87^. tDCS seems therefore to facilitate new learning (rather than consolidate it)^34,88,89^. Interestingly, studies have shown that timing of tDCS application is critical for its effects as well as tDCS is more effective during the encoding phase than the consolidation phase^88,90^; thus tDCS seems to increase task accuracy (especially when baseline is low), and not efficiency^34,91–93^ which is a core feature during visual perception and across attentional networks.

However, Anodal tDCS over the left DLPFC only showed a marginally significant decrease in alerting network efficiency under speed condition when compared to sham stimulation. In this particular case, anodal tDCS seems to have decreased network efficiency for the alerting network, but only under the speed condition. One hypothesis here is that, under speed, response thresholds may be lowered and will depend on more effective brain connectivity^94^. Anodal tDCS has been shown to increase connectivity near the stimulation site, as well to other intra and inter-hemispheric regions^95–97^. However, the effects of tDCS and regions to be affected by it are dependent on the task being performed and the network involved^98^. Furthermore, these task dependency effects may be more important than polarity effects^99,100^. In this sense, it is not the first time that anodal tDCS impairs response times^101^, or that there are no effects of anodal tDCS on RT^102^, or even that tDCS has distinct effects due to the level of expertise when performing the task^103^. Therefore, in order to fully explain these findings, future studies should study how different levels of cognitive load, expertise, even the effects of single site Vs dual site tDCS^104,105^ impacts the oscillatory activity between intra and inter hemispheric regions, and how that is related to performance in order to surpass the physiological effects of tDCS and to better understand the task related effects.

However, this study is not without limitations. First of all, it is not possible to identify if the effects of stimulation are network specific, as ANT performance probably relies on interdependent, rather that independent network performance^106^. Second, in the current design, participants performed the three instruction conditions in a fixed sequence in order to allow for a comparison across stimulation times. Third, it is not possible to disentangle the present results from the motor component of response time, even if our ancillary analysis does not show effects of type of stimulation the RT level per se. Furthermore, the present study does not allow to assess potential differences between low and high frequency tRNS. In fact, previous studies suggested that low and high frequency tRNS may have opposite effects^107^. Or even that only high frequency tRNS was able to increase performance in perceptual learning^78^. However it is also true that the authors did not show statistical significant differences between low and high frequency tRNS^78^, which led other authors to suggest that tRNS effects may indeed be stimulus dependent and as such, lower intensities will induce inhibitory effects (i.e., less than 0.4 mA), while higher intensities will induce excitatory effects (i.e. 1 mA)^108^.

Finally, SAT effects in the present protocol are more evident on the speed condition, however they are not as evident under accuracy condition. As such, the effects of these types of stimulations need to be further explored using tasks with distinct speed and accuracy conditions, which allow a better isolation of the task-dependent networks.

Moreover, studies probing the mechanism of tRNS and tDCS, with varying stimulus intensity and frequencies (in the case of tRNS) using online electroencephalography and/or neuroimaging methods are required to explore stochastic resonance effects through phase synchronization as well as intra and interregional effects of the stimulation.

## Conclusions

Taken together, our current findings showed that tRNS and anodal tDCS over the left DLPFC had differential effects on attention, as measured by the ANT network scores. Overall, we found a recurrent interaction between tRNS and task difficulty in different networks. In the Alerting network, under the Speed instruction, tRNS increased efficiency of the network. Under the more demanding conflict network, tRNS overall increased the performance when comparing to sham. No statistical significant effects of tDCS were observed. These results are compatible with the attention requiring the synchronization of pre-existing networks, rather than the reinforcement or creation of new pathways.
